# Effects of the Litter of Origin and Rearing Litter Size on the Reproductive Potential of Replacement Gilts

**DOI:** 10.3390/ani15203017

**Published:** 2025-10-17

**Authors:** Ryszard Tuz, Martyna M. Małopolska, Tomasz Schwarz, Mirosław Wantuła, Krzysztof Tereszkiewicz

**Affiliations:** 1 Department of Genetics, Animal Breeding and Ethology, Faculty of Animal Science, University of Agriculture in Krakow, Mickiewicza Ave 24/28, 31-120 Kraków, Poland; rztuz@cyf-kr.edu.pl (R.T.); rzschwar@cyf-kr.edu.pl (T.S.); mirekwantula@gmail.com (M.W.); 2 Department of Pig Breeding, National Research Institute of Animal Production, Krakowska 1 Str., 32-083 Balice, Poland; 3 Department of Computer Engineering in Management, Faculty of Management, Rzeszow University of Technology, al. Powstańców Warszawy 12, 35-959 Rzeszów, Poland; kteresz@prz.edu.pl

**Keywords:** gilts, reproductive efficiency, puberty, oestrous cycle, litter size

## Abstract

This study aimed to assess how prenatal and early postnatal conditions affect the future reproductive performance of young female pigs (gilts). Female piglets were selected from sows in their 3rd and 4th parity. Vaginal and cervical length (VCL) was measured using a catheter during the first (VCLI) and second (VCLII) oestrus. Gilts were classified based on their litter of origin: fewer than 16 piglets (OS), 16–18 piglets (OM), and more than 18 piglets (OL). The longest VCLI was found in gilts from the OM group, significantly differing from both the OS and OL groups. For VCLII, significant differences were found between OS and the other groups, with OM and OL showing greater values. Litter size at birth did not negatively affect later reproductive potential. A positive correlation was found between VCLII and the number of piglets in the first litter, indicating that the larger the litter of origin, the higher the reproductive productivity of the gilt. The results highlight that development between the first and second oestrus is critical for vaginal and cervical growth, which, in turn, reflects future reproductive performance. These findings suggest that early life conditions, particularly litter of origin, play a key role in determining the productivity of replacement gilts.

## Simple Summary

There is still a knowledge gap regarding the effect of gilt origin on lifetime reproductive performance. Selecting replacement gilts is often challenging, and each wrong decision negatively affects herd productivity and farm economics. Therefore, the aim of this study was to determine the impact of prenatal conditions on the future reproductive performance of replacement gilts. Starting from the first oestrus, we measured vagina and cervix length (VCL) and analysed the size of the litter of origin. Our results showed that repeated VCL measurements are a reliable tool for predicting reproductive performance. The rate of development varied according to the gilts’ litter of origin just after the first oestrus. Moreover, the highest development rate was observed in gilts originating from the largest litters. In conclusion, replacement gilts should preferably be selected from large litters born to the most prolific dams.

## 1. Introduction

To obtain a high-quality production herd, it is important to focus on the selection of gilts and implementation of appropriate development programmes. The replacement gilts should exhibit first standing oestrus (puberty) at the optimal body weight and age, which guarantees proper development of the reproductive system. The time of puberty has an impact on reproductive potential and sow lifetime productivity [1,2]. Gilts from most genetic lineages reach puberty within 150–210 days of age [3,4] and at around 180–240 days of age are ready for first services. Then, their body weight should be between 135 and 170 kg, depending on breed/commercial lines [5]. Age at puberty may be influenced by many factors, including genetic potential, housing conditions, season, environmental factors, stress, boar exposure, birthweight and management [6,7,8]. Another important trait used to estimate proper development of the reproductive tract is growth rate [9]. Rapid growth of the musculature may inhibit the development of other organs, particularly the reproductive organs. Females with exceptional growth rates are at greater risk of being too heavy, which negatively affects their longevity [9]. Research results have indicated that gilts with a growth rate of 600–700 g/day from birth until first mating tend to produce more piglets at birth than gilts with higher (>700 g/day) and lower (<600 g/day) growth rates [10]. Reproductive traits have low-to-moderate heritability, so testing and selection have hardly improved them [11]. Some traits, such as age at puberty and the weaning-to-oestrus interval, can be used as selection criteria, as they help reduce the number of non-productive days. Nevertheless, focusing on a single trait (e.g., litter size or ovulation rate) is not an efficient way to improve reproduction traits, as the genetic mechanisms of selection have concomitant correlated traits that are often undesirable [7]. However, the litter of sow origin is a factor in which a knowledge gap exists with two opposing theories. First, gilts from small litters should be chosen because the degree of competition among siblings is not high [12,13]. Second, gilts from large litters should be chosen to ensure that daughters have high reproduction potential, similar to that of dams [14]. Another theory focused on rearing conditions exists, where gilts should be chosen from large litters of origin, but the size of the litter should be reduced after 24 h (cross-fostering) to decrease udder competition between piglets [13,15]. Previous research has indicated that vaginal–cervix length (VCL) measurements performed during the first service could be a good tool for predicting a sow’s likely future reproduction efficiency [16]. Furthermore, correlations were found between the ovulation rate and the length of the uterine horn, uterine horn length and VCL, and uterine horn length and uterine capacity [17,18]. The ovulation rate during the first oestrus stage is lower than subsequent ones; thus, mating should be carried out during the second or even third stage of oestrus [19]. Therefore, VCL measurements are usually performed in the second or third heat, when gilts have reached both physiological and breeding sexual maturity. However, a method for selecting replacement gilts as early as possible is needed to reduce rearing costs. One possibility seems to be to corroborate the effect of VCL measurement during the first live oestrus as a potential selection factor. Thus, the objective of this study was to determine the impact of dam prolificacy, which is defined as a litter of origin size that influences the prenatal environmental conditions of the foetus, and a litter of rearing size that influences postnatal environmental conditions, including access to milk, on the reproductive tract development (determined by VCL measurements) and subsequent reproductive efficiency of offspring gilts.

## 2. Materials and Methods

The study was conducted in a commercial facility housing about 320 crossbred (Danish Landrace × Danish Yorkshire) sows located in the Opolskie Voivodeship, Southern Poland. The research was carried out in two separate experiments. The procedures used in the experiment are routine tasks on the farm. Therefore, in accordance with Polish law, our research did not require the approval of the Animal Experimentation Committee.

### 2.1. Experiment 1

The main experimental factor of the first experiment was the effect of the litter of origin on the reproductive ability of gilts. In this experiment, gilts were chosen from litters of different sizes (ranging from 12–21 piglets; mean 17.0 ± 3.2) obtained from 18, 3rd to 4th parity sows. Twenty-four hours after birth (and colostrum intake), gilts were marked by ear tagging and cross-fostered to obtain standardised rearing litters (14.0 piglets per litter). From the 7th day of age, the piglets had access to creep feed, and on day 28, they were weaned and thereafter moved into group pens, with 20 animals per pen. The rearing of the experimental animals was carried out in accordance with standard farm procedures. The selection of gilts was carried out on the basis of structural soundness and condition, and 96 gilts were selected as replacements. The average body weight at first oestrus (mean age 225.1 ± 5.6 days) was 139.6 kg ± 3.7, and that at second oestrus (mean age 246.1 ± 5.2 days) was 152.3 kg ± 3.9.

All the animals received fully balanced dry feed mixes (Table 1) adjusted to their physiological condition and ad libitum access to water. At approximately 14 d prior to mating, flush feeding was provided.

Oestrus was checked daily by an experienced technician via a back pressure test together with vulva observation, and the date of standing heat was recorded. At the time of first-life oestrus occurrence, the vaginal and cervical length (VCL) of each gilt was measured via a foam tip catheter. The first measurement was called VCL I. In the second oestrus period, gilts were inseminated, and another measurement of vaginal and cervical length was carried out (VCL II). The difference between VCL II and VCL I was determined/calculated. Based on the size of the litter of origin, gilts were divided into three groups: those below 16 piglets in the litter (group OS; *n* = 32), those between 16 and 18 piglets in the litter (group OM; *n* = 32), and those above 18 piglets in the litter (group OL; *n* = 32). Ninety gilts were successfully inseminated and gave birth. The mean farrowing rate was 93.75%, which did not differ significantly among the groups. Approximately 7 days before the expected farrowing, the animals were moved to farrowing crates. Next, the number of piglets born in the litter was monitored.

### 2.2. Experiment 2

The main experimental factor in the second experiment was the effect of the litter in which gilts were reared on their reproductive ability, independent of the size of their litter of origin. In this experiment, initially, 96 gilts were chosen. Twenty-four hours after birth and colostrum intake, gilts were marked by ear tagging and separated into three rearing groups. Group RS consisted of 10 piglets per rearing litter, group RM consisted of 12 piglets, and group RL consisted of 14 piglets per rearing litter. Gilts were selected to rearing groups in equal proportions from litters of different sizes of origin to eliminate the potential effect of litters of origin size during further analyses. They were fed according to a two-phase feeding programme (30–110 kg BW and >110 kg BW), similar to experiment 1 (Table 1). Individual body weights were collected at birth and during the first and second life heat stages. The average body weight at first oestrus (mean age 224.9 ± 5.8 days) was 139.6 kg ± 3.6, and that at second oestrus (mean age 246.4 ± 5.4 days) was 152.2 kg ± 4.1.

As in experiment 1, at the time of the first life oestrus occurrence, the vaginal and cervical length (VCL) of each gilt was measured via a foam tip catheter. The first measurement was called VCL I. At the second life oestrus, gilts were inseminated, and another measurement of vaginal and cervical length was carried out (VCL II). The difference between VCL II and VCL I was determined/calculated. Ninety-one gilts were successfully inseminated; however, 1 abortion was noted, which is why 90 gilts gave birth. One week before farrowing, the animals were moved to the farrowing crates, and the number of piglets born during the first farrowing was monitored.

### 2.3. Statistical Analyses

Statistical analyses were performed using SAS 9.2 software (SAS Institute Inc., Cary, NC, USA) to determine the main effects of litter of origin size (<16 vs. 16–18 vs. >18), and litter of rearing size (10 vs. 12 vs. 14). The data were initially subjected to heterogeneity and equal distribution analysis via the Shapiro–Wilk test. The effects of VCL measurements over time according to the litter of origin size or the litter of rearing size were initially analysed using repeated-measures ANOVA. The analyses revealed nonsignificant partial correlation coefficients. Differences among groups in terms of age and body mass during the 1st and 2nd measurements, VCL I, VCL II, differences between VCL II and VCL I, and the number of piglets born in the first parity were assessed by ANOVA followed by multivariate range Duncan’s test. The analyses were performed separately for each experiment because the main experimental factor was different. Pearson’s product-moment test and linear regression were used for correlation analyses of litter of origin size or litter of rearing size with age, body mass, morphometric markers of reproductive tract development (VCL I, VCL2 II, and the difference between VCL II and VCL I) and the first litter size. Additionally, associations between the measured morphometric markers (VCL I, VCL2 II, and the difference between VCL II and VCL I) and the first litter size were specified as Pearson correlation. Statistical significance was declared at *p* value < 0.05 and *p* ≤ 0.01 in all analyses. All the results are presented as the means ± standard deviations (SD).

## 3. Results

During the analyses of the litter of origin effect, the largest VCL I was found in gilts from the OM group, and the difference was significant (*p* < 0.05) in comparison with the OS and OL groups, despite the small numerical difference. The VCL II was significantly different (*p* < 0.01) between the OS group and the other analysed groups. This was caused mainly by the advantage of the OM group in VCL I and the significantly greater increase in vaginal and cervical length between measurements in the OL group than in the OS (*p* < 0.01) and OM (*p* < 0.05) groups. A similar relationship (*p* < 0.01) was found for the number of piglets born in the first litter. The significantly larger litters were obtained from gilts in the OL group than from females in the OS and OM groups (Table 2). Moreover, there were no significant differences in gilt age or body weight among the groups. The Pearson correlation coefficient between body weight and VCL measurement in the first oestrus was −0.173 (NS), and in the second oestrus −0.081 (NS).

Repeated-measures ANOVA showed significant within-subject effects in the univariate test of time × VCL and time × VCL × litter of origin size (Table 3). The significant effect of time × VCL × litter of origin size was visible in the OL group, where the difference between VCLII and VCLI was significantly greater than that in the other groups (Table 2).

During analyses of the litter rearing effect, the largest VCL I (*p* < 0.01) and VCL II (*p* < 0.05) values were observed in the RL group, in comparison to the RS and RM groups, whereas a greater VCL difference was noted in the RM group. Despite this, the VCL difference was not statistically significant, nor was the difference in the number of piglets born in first parity (Table 4). Similar to experiment 1, no significant differences were found between the groups in terms of gilts’ age or body weight. The Pearson correlation coefficient between body weight and VCL was 0.088 (NS) in the first oestrus, and 0.086 (NS) in the second oestrus.

Repeated-measures ANOVA revealed a significant within-subject effect of time × VCL in the univariate test but a nonsignificant effect of time × VCL × litter size (Table 5). This was due to the lack of a significant difference between the VCLII and VCLI in any of the analysed groups (Table 4).

The time_VCL effect was significant in both experiments, with a significant increase in VCL measurements between timepoints in all analysed populations. The Time_VCL × litter effect was significant in experiment 1, but only in one group—OL (gilts born in the largest litter of origin). The Time_VCL × litter effect was not significant in experiment 2. The results of the two-way ANOVA for the main effects of litter of origin and litter of rearing in Experiment 2 are presented in Supplementary File Table S1. These additional analyses confirm that postnatal rearing conditions (litter of rearing) exerted only a limited influence on reproductive parameters, with a significant effect detected exclusively in VCL1.

There was no significant correlation between litter-of-origin and VCL I, while there were significant (*p* < 0.01) positive relationships between litter-of-origin and VCL II, VCL difference, and the number of piglets in the first parity, with a moderate correlation. Moreover, there were no significant correlations between the rearing size of the litter and any of the analysed parameters. VCL I and the number of piglets in first parity were not significantly correlated, whereas a significant (*p* < 0.01) positive relationship was found between VCL II and the number of piglets in first parity. A significant correlation (*p* < 0.01) was also noted between the VCL difference and the number of piglets in the first parity (Table 6).

## 4. Discussion

Early detection of the onset of puberty is important for mating sector management, which also influences herd productivity [20]. Kapelański et al. [21] noted the occurrence of significant differences in the size of the reproductive tract of gilts; however, they did not determine when this differentiation occurred. In our research, strict control over the appearance of first-life oestrus was established, and after its detection, measurement of VCL was conducted. Due to the fact that gilts of the analysed breed are fast-growing, breeding maturity was achieved in the second life oestrus, and then VCL measurements (VCL II) were performed during insemination. The relationships among the litter of origin, the development of the reproductive system and the production potential are interesting. A range of environmental and physiological conditions experienced by sows during gestation may modulate the developmental programming of their litters, thereby affecting subsequent growth, metabolic, and reproductive outcomes [22,23,24]. Both the litter of origin and the environmental conditions to which pigs are exposed during prenatal and postnatal development exert significant influences on their physiological programming. While the litter of origin determines the initial framework of reproductive and metabolic potential through genetic and intrauterine factors, environmental conditions—such as maternal nutrition, housing, and management practices—can either exacerbate or mitigate these early-life effects, ultimately shaping growth, reproductive performance, and lifetime productivity [25,26,27]. Analysis of reproductive tract growth, determined by the difference between VCL II and VCL I measurements, revealed a significant relationship with the number of piglets born at first parity. These relationships were confirmed by statistically significant coefficients of correlation. Flowers [28] reported that over 50% of the variation in lifetime productivity for sows (total R^2^  =  0.51) is associated with variation in the litter of origin traits. Ji et al. [29] noted that any adverse conditions occurring during gestation or nursing have a negative impact on offspring, can last throughout their life cycle and can be carried over to the next generation or beyond. Thus, the litter of origin (prenatal programming) contains a significant proportion of variation in gilt development [7,30]. Warda et al. [31] noted that sows from small litters of origin (≤9) were the oldest at the start of reproductive life and that they were the first to be removed from the herd, which was responsible for their poorer performance during productive life. Our results indicated that the larger the litter of origin was, the greater the productivity of gilts. Consequently, the litter of origin is important for the productivity of replacement gilts.

Another important feature is birth weight; gilts born to low-birth-weight sows can pass this trait on to their offspring [9,32]. Additionally, in some analyses, smaller gilts had a smaller uterus and less uterine secretion than larger female pigs did [33,34,35]. However, in our results, there was no significant correlation between body weight and VCL measurement in either the first or the second heat. Previous research results indicated that gilts raised in small litters reached puberty earlier and subsequently improved in terms of retention in the breeding herd compared with those raised in large litters [13]. This finding appears contradictory to the observations of Warda et al. [31]. These differences may arise from variation in study populations, management conditions, or definitions of litter size categories. While earlier puberty may favour a breeding herd, the long-term performance and culling risk can still be negatively affected by reduced prenatal growth or limited maternal investment typical of small litters. Thus, both sets of results suggest that the litter of origin exerts complex, multifactorial effects on gilt development and lifetime productivity, which may manifest differently depending on environmental and genetic contexts. In gilts weaned from small litters, positive relationships were identified between preweaning growth rates and early age at puberty and between the number of pigs weaned and the numbers of primordial, primary, and antral follicles [7]. Our results revealed no effect on the productivity of replacement gilts according to the size of the rearing litter, which ranged from 10 to 14 pigs. The low and statistically insignificant coefficient of correlation indicates the lack of a linear relationship between the size of the litter reared and the development of the reproductive system of the gilt at the time of puberty (VCL I, VCL II, and the difference between VCL II and VCL I). These results suggest that the litter of origin has a more substantial effect on VCL measurements and reproductive performance than does the litter of rearing. Flowers [28] reported that preweaning growth and weaning weights probably reflect the suitability of the early postnatal environment for supporting reproductive tract development in much the same way that birthweights are related to the adequacy of prenatal conditions. The presented data seem to indicate that the key period of vaginal and cervical development in gilts is the first oestrous cycle, while the pace of this development is closely related to the reproductive potential of dams. The explanation of this process mechanism requires further research, but the current state of knowledge allows us to make an explanatory hypothesis. In pigs, the highest inherited reproductive trait is the ovulation rate, which has been shown in many studies [36,37]. Therefore, it should be expected that daughters of dams with a high level of ovulation will be characterised by increased follicular function of the ovaries and thus an increased concentration of oestradiol in the blood serum [6,38]. Oestradiol is a key hormonal factor that is responsible for uterine growth and development [39]. Oestrogen enables uterine proliferation (the length of the uterine horns and the number of uterine glands), which depends on the synthesis of IGF1 growth factor [40]. This seems to clarify the straightforward relationship between ovarian function in the first oestrus and the tempo of reproductive tract development during the first oestrous cycle. These findings were confirmed in our study by the difference between VCL II and VCL I among gilts originating from litters of different sizes. The final confirmation was the differences in the first litter size among the groups.

## 5. Conclusions

This study demonstrates that the litter of origin significantly influences the reproductive development and future productivity of replacement gilts. Gilts born into larger litters exhibit a more rapid increase in vaginal and cervical length between their first and second oestrous cycles—an indicator likely linked to enhanced ovarian activity, increased ovulation rates, and elevated oestradiol levels. While this hypothesis requires further validation through hormonal and ovarian function analyses, the observed trend highlights the physiological advantages conferred by a more competitive prenatal and postnatal environment.

Importantly, the second measurement of vaginal and cervical length (VCL II), taken during the second oestrous cycle, proved to be the most reliable predictor of subsequent reproductive performance. In contrast, the initial measurement (VCL I) was not a dependable indicator, as gilts from large litters often showed low VCL I values but experienced substantial development thereafter. The change between the first and second measurements provided some predictive insight but was less effective than the VCL II measurement alone.

These findings are highly relevant for improving gilt selection strategies in commercial pig production. By identifying physiological markers such as VCL II that correlate with reproductive potential, producers can make more informed decisions when selecting replacement gilts, ultimately enhancing herd fertility and productivity. Additionally, the results underscore the broader significance of early-life conditions—particularly litter size at birth—in shaping long-term reproductive outcomes in pigs.

## Figures and Tables

**Table 1:** Gilts’ diet formulation and chemical composition.

Diet Composition	30–110 kg	>110 kg
Winter barley (%)	28.8	30
Triticale (%)	27	26
Wheat (%)	20	15
Wheat bran (%)	5	20
Soybean meal–46%CP (%)	14.5	5.8
Vitamin–mineral premix (%)	3.0	2.5
Limestone (%)	1.5	0.30
Soybean oil (%)	0.2	0.40
Daily feed intake (kg/day)	2.8–3.0	3.0–3.5
**Nutrients:**		
Metabolizable energy (ME, kcal/kg)	3.200	3.050
Crude protein (%)	16.5	14.5
Lysine (%)	0.85	0.70
Calcium (%)	0.65	1.0
Total phosphorus (%)	0.55	0.55
Crude fibre (%)	4.5	5.5

**Table 2:** VCL measurements and number of piglets born in first parity from gilts of different litters of origin in experiment 1.

Item	The Size of the Litter of Origin (Unit)		
	Group OS <16	Group OM 16–18	Group OL >18
	Mean ± SD	Mean ± SD	Mean ± SD
*n*	30	31	29
Litter of origin	13.20 ^A^ ± 0.75	17.00 ^B^ ± 0.89	20.80 ^C^ ± 0.40
Litter of rearing	14	14	14
VCL I (cm)	17.60 ^Aa^ ± 1.22	18.63 ^Bb^ ± 1.67	17.77 ^ABa^ ± 1.97
VCL II (cm)	19.30 ^A^ ± 0.92	21.30 ^B^ ± 1.31	21.87 ^B^ ± 2.26
difference between VCL II and VCL I (cm)	1.70 ^Aa^ ± 1.45	2.67 ^ABb^ ± 1.80	4.10 ^Bc^ ± 2.90
Number of piglets in first parity	15.13 ^A^ ± 2.13	16.00 ^A^ ± 2.60	17.73 ^B^ ± 1.55

^a,b,c^ Values within a row with different superscripts differ significantly at *p* < 0.05. ^A,B,C^ Values within a row with different superscripts differ significantly at *p* < 0.01.

**Table 3:** Repeated-measures ANOVA univariate test of hypotheses for within-subject effects in experiment 1.

Source	DF	Type III SS	Mean Square	F Value	Pr > F
time_VCL	1	359.4533638	359.4533638	174.06	<0.0001
time_VCL × litter	2	43.7338771	21.8669386	10.59	<0.0001
Error(time_VCL)	87	179.6605673	2.0650640		

**Table 4:** VCL measurements and number of piglets born in first parity from gilts of differential litters of rearing in experiment 2.

Item	The Size of the Litter of Rearing (Unit)		
	Group RS 10	Group RM 12	Group RL 14
	Mean ± SD	Mean ± SD	Mean ± SD
*n*	29	32	29
Litter of origin	17.07 ± 3.17	16.60 ± 3.42	17.33 ± 2.89
VCL I (cm)	17.67 ^A^ ± 1.72	17.60 ^A^ ± 1.50	18.73 ^B^ ± 1.57
VCL II (cm)	20.33 ^a^ ± 1.98	20.73 ^ab^ ± 1.78	21.40 ^b^ ± 1.85
difference between VCL II and VCL I (cm)	2.67 ± 2.73	3.13 ± 2.06	2.67 ± 2.08
Number of piglets in first parity	16.47 ± 2.45	15.87 ± 2.65	16.53 ± 1.86

^a,b^ Values within a row with different superscripts differ significantly at *p* < 0.05. ^A,B^ Values within a row with different superscripts differ significantly at *p* < 0.01.

**Table 5:** Repeated-measures ANOVA univariate test of hypotheses for within-subject effects in experiment 2.

Source	DF	Type III SS	Mean Square	F Value	Pr > F
time_VCL	1	355.6906994	355.6906994	128.24	<0.0001
time_VCL × litter	2	1.2724168	0.6362084	0.23	0.7955
Error(time_VCL)	87	241.3053610	2.7736248		

**Table 6:** Summary of correlations between analysed traits.

Input Variable (x)	Output Variable (y)	Regression Equation	*p* Value	r ^1^
Litter-of-origin	VCL I	y = 0.015x + 17.74	0.698	0.029
Litter-of-origin	VCL II	y = 0.34x + 15.04	0.000000	0.563
Litter-of-origin	VCL difference	y = 0.32x − 2.695	0.00001	0.445
Litter-of-origin	Number of piglets/first parity	y = 0.34x + 10.47	0.000005	0.461
Litter-of-rearing	VCL I	y = 0.15x + 16.02	0.064	0.139
Litter-of-rearing	VCL II	y = 0.15 + 18.84	0.105	0.121
Litter-of-rearing	VCL difference	y = 0.000x + 2.82	1.000	0.000
Litter-of-rearing	Number of piglets/first parity	y = 0.010x + 16.17	0.935	0.006
VCL I (cm)	Number of piglets/first parity	y = 0.22x + 12.39	0.147	0.154
VCL II (cm)	Number of piglets/first parity	y = 0.85x − 1.43	0.000000	0.692
difference between VCL II and VCL I (cm)	Number of piglets/first parity	y = 0.47x + 14.96	0.000005	0.461

^1^ coefficient of correlation.

## Data Availability

None of the data were deposited in an official repository. The datasets are available from the corresponding author upon reasonable request.

## Supplementary Materials

The following supporting information can be downloaded at: https://www.mdpi.com/article/10.3390/ani15203017/s1, Table S1: Summary of two way ANOVA for main effects of litter of origin and litter of rearing in experiment 2.

## Abbreviations

The following abbreviations are used in this manuscript:

VCL I	Vaginal and cervical length measured via catheter during the first oestrous cycle
VCL II	Vaginal and cervical length measured via catheter during the second oestrous cycle
OS	Litter of origin with fewer than 16 piglets
OM	Litter of origin with 16 to 18 piglets
OL	Litter of origin with more than 18 piglets
RS	Litter of rearing with 10 piglets
RM	Litter of rearing with 12 piglets
RL	Litter of rearing with 14 piglets
