# Supplementary material for: Effects of the Litter of Origin and Rearing Litter Size on the Reproductive Potential of Replacement Gilts
Source: Animals (Basel). 2025 Oct 17;15(20):3017. doi: 10.3390/ani15203017 (PMC12560926; doi:10.3390/ani15203017)
Supplement: Supplementary file 1 [file animals-15-03017-s001.zip › animals-3899363-supplementary.pdf]

Table S1. Summary of two way ANOVA for main effects of litter of origin and litter of rearing in experiment 2

Source	DF	SS	Mean Square	F Value	Pr > F
			VCL 1		
VCL1 free range	1	27532.75	27532.75	10840.02	<0.0001
Litter of origin	2	12.58	6.29	2.48	0.0904
Litter of rearing	2	18.57	9.29	3.66	0.0301
Litter of origin × litter of rearing	4	12.12	3.03	1.19	0.3203
Error	81	205.73	2.54		
			VCL 2		
VCL2 free range	1	36755.64	36755.64	14219.58	<0.0001
Litter of origin	2	104.42	52.21	20.20	<0.0001
Litter of rearing	2	12.28	6.14	2.38	0.0993
Litter of origin × litter of rearing	4	1.19	0.30	0.12	0.9768
Error	81	209.37	2.58		
			difference between VCL II and VCL I		
VCLdiff. free range	1	665.00	665.00	139.77	<0.0001
Litter of origin	2	81.61	40.80	8.58	0.0004
Litter of rearing	2	4.88	2.44	0.51	0.6008
Litter of origin × litter of rearing	4	7.88	1.97	0.41	0.7982
Error	81	385.37	4.76		
			Number of piglets in first parity		
No of piglets in 1st parity free range	1	22444.11	22444.11	5082.06	<0.0001
Litter of origin	2	114.01	57.00	12.91	<0.0001
Litter of rearing	2	7.93	3.97	0.90	0.4114
Litter of origin × litter of rearing	4	31.06	7.77	1.76	0.1453
Error	81	357.72	4.42		
